# Healthcare Awareness Profile Interview: Development of a new evidence-based brief clinical tool to assess awareness in people with dementia

**DOI:** 10.1080/09602011.2024.2337152

**Published:** 2024-04-17

**Authors:** Catherine M. Alexander, Anthony Martyr, Linda Clare

**Affiliations:** a REACH: the Centre for Research in Ageing and Cognitive Health, University of Exeter Medical School, University of Exeter, Exeter, UK; b National Institute for Health and Care Research (NIHR) Applied Research Collaboration, South-West Peninsula, UK

**Keywords:** Alzheimer’s disease, Anosognosia, Insight, Measure development, Post-diagnostic support

## Abstract

People with dementia vary in awareness of difficulties. Evaluating awareness could facilitate personalized care. However, current research measures are unsuitable for practical clinical application. We aimed to develop a brief multidimensional awareness interview for clinical use. Informed by available evidence about awareness of dementia, items suitable for both in-person and remote administration were modified from validated measures or developed for clinical application. The interview was administered via telephone or videoconference to 31 community-dwelling people with mild-to-moderate dementia. An informant completed a corresponding questionnaire. A multidimensional profile of awareness was created using self-report of symptoms, and discrepancies between self-rating and either informant rating or objective memory task performance. Feedback from participants and informants and discussions with clinical advisory and patient and public involvement groups helped finalize the interview. Remote administration was straightforward taking on average under 11 min. Awareness profiles showed a spectrum of awareness across domains. Feedback indicated that the items were acceptable and understandable. Certain aspects could be mildly upsetting where current difficulties were highlighted. Subject to further validation, the Healthcare Awareness Profile Interview (HAPI) shows potential as an evidence-based brief clinical tool for assessing awareness in people with mild-to-moderate dementia.

## Introduction

People with dementia vary in awareness about their condition and the difficulties that dementia entails (Alexander et al., 2022; Alexander, Martyr, Gamble, et al., 2021; Villarejo-Galende et al., 2022). Differences can also be seen in awareness or self-appraisal of functioning on everyday cognitive tasks and functional activities, and socioemotional functioning (Clare, Whitaker, et al., 2011). The consequences of having either high or low awareness of their difficulties are significant for people with dementia (Alexander, Martyr, Gamble, et al., 2021; Azocar et al., 2021; Starkstein et al., 2007) and can impact their carers (Alexander et al., 2023; Clare, Whitaker, et al., 2011; Nelis et al., 2011; Turró-Garriga et al., 2013). Understanding awareness in people with dementia could become a valuable part of clinical practice around and after diagnosis (Clare, Marková, et al., 2011; Lacerda et al., 2020). Increased awareness of difficulties is related to lower mood (Azocar et al., 2021) and lower perceived ability to live well for the person with dementia (Alexander, Martyr, Gamble, et al., 2021; Martyr et al., 2019). Being less aware of difficulties can lead to unsafe decisions (Parrao et al., 2017; Starkstein et al., 2007), and more stress for carers (Alexander et al., 2023; Nelis et al., 2011; Turró-Garriga et al., 2013). The degree of awareness has been shown to influence involvement in decision-making (de Souza et al., 2022; Karlawish et al., 2005) and impact health and social care outcomes (Parrao et al., 2017).

Awareness can change in mild-to-moderate dementia (Alexander et al., 2022) and this might shape how, when, and what support should be provided. Assessing and monitoring changes in awareness would allow phased communication and targeted support, depending on the specific level of awareness a person has. Understanding awareness could also enhance clinical communication around issues other than dementia, facilitating optimal involvement in decisions about healthcare (de Souza et al., 2022; Karlawish et al., 2005).

The degree of awareness varies for different aspects of everyday function, sometimes described as different “objects” of awareness (Marková et al., 2014). This means for example that awareness of memory impairment may diverge from awareness of difficulties in activities of daily living (Lacerda et al., 2020; Marková et al., 2014). Recently, a change over time in awareness of activities of daily living, as shown in self-ratings, was found to be more consistent with the change in objective cognition than was the case for informant ratings of the same activities (Martyr et al., 2024). Awareness can operate at different levels of processing; for instance, awareness of problems with memory performance during a task may differ from awareness when reflecting on memory function at an evaluative level (Clare, Marková, et al., 2011). Differing amounts of awareness at these different levels can influence the modification of behaviour (Shaked et al., 2019) and/or emotional responses to situations (Mograbi, Brown, et al., 2012).

The most common method of measuring awareness in research uses the discrepancy between self-ratings by the person with dementia and ratings by an informant, usually the carer, as an indication of impaired awareness (Alexander, Martyr, Savage, et al., 2021). However, sole reliance on this method has limitations due to the potential inaccuracy of informant ratings (Martyr & Clare, 2018). Clinician ratings and semi-structured interviews can provide information about awareness, but can be subjective (Alexander, Martyr, Savage, et al., 2021). Other methods include comparing self-ratings with objective performance (Alexander, Martyr, Savage, et al., 2021) or the use of self-report alone (Alexander et al., 2022; Alexander, Martyr, Gamble, et al., 2021; Mayelle et al., 2019; Villarejo-Galende et al., 2022). The combined use of different methods can assess different aspects of awareness and provide more comprehensive information (Clare, Whitaker, et al., 2011).

There is no gold standard for measuring awareness in research, and no established method of assessing awareness clinically (Alexander, Martyr, Savage, et al., 2021). In our review many measures aimed to categorize people according to lack of awareness, assuming it to be a unified construct (Alexander, Martyr, Savage, et al., 2021). However, existing measures have shortcomings when considered for person-centred clinical use. For example, some have very specific aims such as risk assessment or are limited to a single object of awareness, typically memory, or only use self/informant-rated discrepancies. A structured approach avoids reliance on individual clinical judgement, but research measures can be too complex and lengthy for clinical use. A multidimensional clinical measure of awareness designed to support care that is tailored to the individual needs of people with dementia would be useful for several reasons. A new tool could be used by clinicians who provide post-diagnostic care, i.e., professionals from the multidisciplinary team working in memory clinics or primary care, as a way to gather information, start conversations, document the current position concerning awareness, map changes in awareness, and provide a shared language to describe awareness issues. A formal awareness assessment could record awareness of dementia-related difficulties, facilitating further discussions about future treatment and care. The assessment could include clinically important areas like awareness of medication management and awareness of own health symptoms where these are relevant for the individual, as well as assessing awareness regarding other instrumental activities of daily living (iADL), memory, and socioemotional functioning, to assist in clinical communication and provision of appropriate support.

### Aims and objectives

The overall aim was to develop and test a new tool for use in a clinical setting to measure awareness in people with mild-to-moderate dementia, either in a face-to-face consultation or by telemedicine. A review of existing research and consultation with experts by experience and clinicians guided item selection and the structure of the tool. A pilot study was designed to test the acceptability of items and the administrative feasibility of the tool, to optimize the tool, named the HAPI, for subsequent validation.

## Materials and methods

### Proposed format of the tool and item selection

The format and item selection were guided by research evidence showing the importance of assessing awareness across different objects and using a range of methods. Selected items were modified for use in a clinical tool, incorporating features suggested in a scoping review (Alexander, Martyr, Savage, et al., 2021), i.e., brief, clinically relevant, and supportive of person-centred care, and feasible for administration in a structured interview conducted either face-to-face or remotely by telephone or videoconferencing.

### Consultation with patient and public involvement (PPI) group and clinical advisory group

The initial version of the tool was shared with a PPI group comprising people with dementia and carers, and a clinical advisory group comprising a consultant geriatrician, a general practitioner, and a senior memory nurse, all with clinical and academic roles in the South West of England.

### Pilot study

#### Study design

The study was designed to test the feasibility and acceptability of the tool and was conducted remotely due to contemporaneous coronavirus restrictions. The awareness interview was administered by telephone or videoconference to the participants at home. Feedback questions for participants and informants were interspersed to assess the ease of understanding and whether any upset was experienced.

Ethical approval for the pilot study was given by the University of Exeter College of Medicine and Health Research Ethics Committee reference Aug20/B/244.

#### Participants

People of any age or sex, with a clinical diagnosis of dementia of any subtype, were enrolled if they lived at home and had an available informant. The diagnosis was obtained either from the Join Dementia Research (JDR; http://www.joindementiaresearch.nihr.ac.uk/) online portal or from records available to the team where participants had taken part in previous research studies; for two participants this information was volunteered by the informant. Both needed to have adequate hearing and be able to manage a structured interview by telephone or videoconference. The target sample size was 25 dyads, building on the minimum recommendations for studies to test the wording and formatting of an instrument (Hertzog, 2008). People who self-defined as having mild or moderate dementia were contacted to take part. This self-definition was corroborated by a target score between 11 and 30 on a cognitive screening test, the Montreal Cognitive Assessment Five-minute protocol (MoCA-5 min; Wong et al., 2015). As the MoCA-5 min was completed at the end of the study due to study design requirements, data from participants with cognitive scores below the target range were included in the analysis if participants were able to manage the interview satisfactorily with responses documented to most or all items. See Supplementary Text for further details of exclusion criteria.

#### Recruitment

Recruitment and subsequent interviews took place in England between October 2020 and November 2021. Participants were mainly recruited via the Join Dementia Research online portal (JDR). Recruitment also took place from a local memory café following a presentation about the research at a virtual meeting. In addition, willing participants who had been involved in previous studies conducted by the research team were invited to take part.

#### Demographic details

Basic demographic details were reported by participants and informants; see Supplementary Text for details.

#### Measures

##### Awareness interview

The informant completed a written questionnaire with nine items, each requiring a response using a five-point scale. The informant was asked to rate the ability or aptitude of the participant in four areas: mobility, functional abilities (iADL), everyday memory, and socioemotional functioning. For the participants, questions were posed in a structured interview containing 13 items. For 10 items, responses used a five-point scale, and a colour response chart was provided to facilitate the selection of response options during the interview. Nine of these items corresponded to the items in the informant questionnaire. The participant was also asked to undertake a short memory task involving immediate story recall, followed by self-evaluation of performance on a five-point scale. In addition, the participant answered three questions that assess awareness of typical dementia symptoms/condition.

##### Cognitive assessment

The MoCA-5 min (Wong et al., 2015) was administered to the participant after the awareness interview to avoid influencing awareness of memory problems. Total scores range from 0 to 30 with higher scores indicating better cognitive function.

##### Feedback questions

Structured feedback using five-point scales was obtained from participants and informants during and after the interview. Additional comments were invited after the story recall task, and about the overall experience of the interview. See Supplementary Text for more details.

##### Field notes

Brief written notes documented issues arising during the administration of the tool and any comments or reactions that were not reported in formal feedback responses.

#### Procedure

The informant questionnaire was sent by post, along with the colour response chart for the participant to use during the interview; see Supplementary Figure 1. The informant was asked to complete the informant questionnaire independently prior to the meeting. Consent was audio recorded. The responses to the informant questionnaire were relayed verbally to the researcher during the interview, for example, “Question 1: Response a),” without elaboration of the questions. The informant was invited to remain in the room during the participant interview unless the participant requested otherwise. It was emphasized that the responses during each interview needed to be given independently, with no conferring or prompting.

#### Analyses

Interview item responses were analysed to show the range and frequency of responses and frequency of non-responses. Scores were created for each item and converted to awareness bands for each section as described in the proposed scoring; see Supplementary Text.

The structured feedback responses were collated to assess individual and group reactions to items, looking particularly at the frequency of responses where an item was rated difficult or upsetting. The free-text responses were analysed using quantitative content analysis (Rose et al., 2014; White & Marsh, 2006). Negative responses to items and findings from the field notes were compiled to identify significant or recurrent issues about items or administration that might inform further development and finalization of the tool.

### Revisions and finalization of the tool

#### Identifying issues to review

Issues requiring review arose from participant or informant feedback in the study, examination of item performance, or observations recorded in the field notes. Other issues for planned review from the tool development stage were consideration of item reduction, and review of the proposed scoring.

#### Group discussions

Preliminary discussions took place within the research team, followed by review meetings with the PPI group and the clinical advisory group.

## Results

### Item selection for the pilot version of the awareness tool

Item selection is described briefly below, with more detail in the Supplementary Text and Supplementary Table 1. Analysis of secondary data (Clare et al., 2012) allowed selection of items with high item-total correlation from the Memory Awareness Rating Scale (MARS; Clare et al., 2002), the modified Functional Activities Questionnaire (FAQ; Martyr et al., 2012; Pfeffer et al., 1982) and the Socio-Emotional Questionnaire (SEQ; Bramham et al., 2009), to assess awareness of memory, functional activity and socioemotional ability.

Examination of item response patterns in existing data (IDEAL; Clare et al., 2014) led to the selection of three items from the screening checklist from the Representations and Adjustment to Dementia Index (RADIX; Quinn et al., 2018), measuring acknowledgement of difficulties associated with dementia, considered as awareness of condition (Alexander et al., 2022; Alexander, Martyr, Gamble, et al., 2021).

Other validated measures of iADL (Fillenbaum, 1988; Patterson et al., 1992) and health status (The EuroQol Group, 1990) were reviewed to inform the development of items to assess awareness of medication management and health symptoms other than dementia, using self-rating/informant discrepancy.

#### Awareness of memory performance and function

Taken from the MARS memory performance scale, the story’s immediate recall item was selected. The story used with the MARS is subject to copyright. An alternative story that has been used in a similar way is the Babcock story recall test (Lezak et al., 2012), and was incorporated into the new tool. The story recall item assesses the ability to recall a short news item immediately after it is presented. The person gives an evaluation of his/her performance which can be compared to the objective score. Calculation of a discrepancy between the self-evaluation and objective performance is used as an indication of awareness of memory performance. A corresponding item was selected from the MARS memory function scale. This radio recall item compares self-rating of general ability to recall a radio-news story with informant rating, using the discrepancy to indicate awareness of memory function.

#### Awareness of functional ability

Items selected from the modified FAQ were about shopping alone and using a telephone independently, both adapted and used to calculate the discrepancy between self – and informant ratings of these iADL tasks. A new question was formulated about independent medication management, also designed to calculate a discrepancy score between self- and informant ratings. Regular prescribed medication is not universal; therefore, this item is only administered if it is reported by the participant. If the participant does not know, or the participant or informant reports no regular medication, this item is not included in the scoring.

#### Awareness of socioemotional functioning

From the SEQ, items were selected comparing self-rating of socioemotional functioning with an informant rating. The selected items were “when others are sad I comfort them,” “I am confident meeting new people,” and “I avoid arguments.” An additional item requiring reversed scoring was included to see if negatively phrased items would be a useful contribution: “I am impatient with other people.”

#### Awareness of condition

The three RADIX screening items selected to identify people with low awareness of the condition were about noticing difficulties with being forgetful, with concentration, and with the ability to say what you want to say. Compared to using the full RADIX nine-item checklist (Alexander, Martyr, Gamble, et al., 2021), the three selected items matched the categorization of 93% of people judged as showing some awareness when the full checklist was used.

#### Awareness of physical condition: Mobility

A new item about awareness of mobility/being able to walk about safely was designed as a self-rating/informant discrepancy item, using a five-point response scale in line with the other selected items for the new tool. Mobility was chosen as it has relevance to many people with dementia.

### Consultation with PPI group and clinical advisory group

The proposed format of the tool was shared with the PPI and clinical advisory groups, leading to some minor amendments to the wording and ordering of the items.

### Pilot study

#### Recruitment, demographic information, and interview details

**Figure 1.**
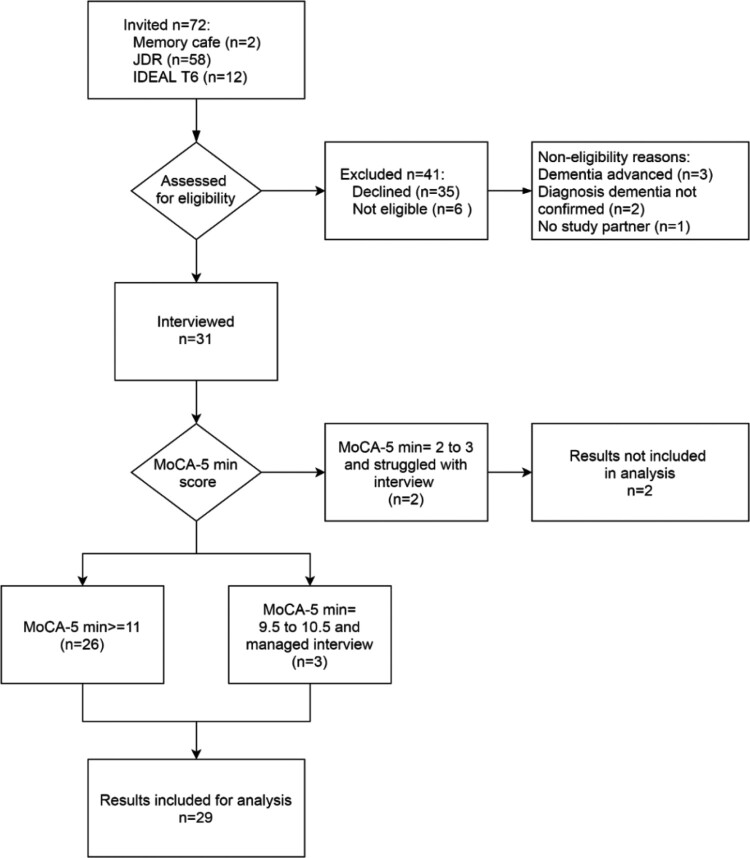
Flowchart for participant recruitment.

There were 31 participants recruited, mainly from JDR; see Figure 1. Two people had difficulty completing the interview with a considerable number of non-responses. These people were found to have substantially lower cognitive scores than the other participants (MoCA-5 min scores 2 and 3). Their results were therefore not included in the analyses. Three people with MoCA-5 min scores of 9.5–10.5 were just below the target MoCA-5 min lower range of 11 and managed the interview satisfactorily. The results for these three people were retained in analyses. See Table 1 for the demographic information for the 29 dyads included in the analyses. On average the awareness interview lasted approximately 11 min, excluding the feedback questions and time for collecting the informant responses; see Table 2 for details.

**Table 1.** Demographic characteristics of participants and informants.

Characteristic	Participants with dementia (*n* = 29)	Informant (*n* = 29)
Age years: mean (SD); median (range)	75.24 (7.19); 76 (54–86)	70.10 (10.50); 72 (44–88)
Age group n (%)		
<65y	3 (10.3)	7 (24.1)
65–69y	2 (6.9)	4 (13.8)
70–74y	7 (24.1)	9 (31.0)
75–79y	8 (27.6)	4 (13.8)
80+ y	9 (31.0)	5 (17.2)
Sex *n* (%)		
Male	18 (62.1)	7 (24.1)
Female	11 (37.9)	22 (75.9)
Ethnicity *n* (%)		
White British	25 (86.2)	27 (93.1)
White other	3 (10.3)	2 (6.8)
Black African	1 (3.4)	–
Dementia diagnosis *n* (%)		
Alzheimer’s disease	12 (41.4)	
Vascular dementia	4 (13.8)	
Mixed dementia	7 (24.1)	
Dementia with Lewy bodies	3 (10.3)	
Frontotemporal dementia	3 (10.3)	
Length of time since diagnosis *n* (%)		
<1y	4 (13.8)	
1–2y	12 (41.4)^†^	
3–5y	6 (20.7)^‡^	
6+ y	5 (17.2)	
Missing	2 (6.9)	
MoCA-5 min score: mean (SD); median (range)	19.97 (5.82); 19.0 (9.5–29.0)	
Education		
Age left school years: mean (SD); Median (range)	16.03 (1.24); 16 (13–18)	16.10 (1.35); 16 (14–19)
Highest qualification: n (%)		
No formal qualifications	4 (13.8)	2 (6.9)
Age 16 school leaving certificate	13 (44.8)	9 (31.0)
Age 18 school leaving certificate	7 (24.1)	11 (37.9)
University qualification	5 (17.2)	7 (24.1)
Relationship to participant *n* (%)		
Spouse/partner		25 (86.2)
Daughter/son		4 (13.8)
Length of relationship spouse/partner in years: mean (SD); median (range)		44.72 (15.01); 48 (11–68)
Length of relationship daughter/son in years: mean (SD); median (range)		50.50 (5.51); 51 (44–56)
Living arrangement *n* (%)		
Informant lives with participants		26 (89.7)
Informant lives elsewhere		3 (10.3)
If lives elsewhere, number of days contact per week *n* (%)		
7		1 (3.4)
4		1 (3.4)
2		1 (3.4)

Abbreviation: MoCA-5 min, Montreal Cognitive Assessment Five-minute protocol.

†Includes estimated from Join Dementia Research registration (*n* = 3).

‡Includes estimated from Join Dementia Research registration (*n* = 1).

**Table 2.** Awareness interview details.

Mode and situation of interview	*n* (%)
Mode of interview *n* (%)	
Telephone	7 (24.1)
Videoconference	22 (75.9)
Interview situation *n* (%)	
Participant interview with an informant in the same room	24 (82.8)
Participant interview with an informant in a different room	5 (17.2)
Informant completed responses in advance	26 (89.7)
Informant completed responses with researcher; participant in different room	3 (10.3)
Time recorded for participant awareness interview in minutes: mean (SD); median (range); missing	All 10.94 (2.67) Telephone 10.38 (2.69) Videoconference 11.14 (2.70) 10.53 (7.67–18.23); 2

#### Awareness interview results

##### Response rates

The response rate was high, with three non-responses to individual items. There was no apparent demographic pattern for non-responders. For the medication-independent item, the discrepancy could not be calculated for three participants. This was due to non-administration of the item to two participants who, when asked if they took regular medication, reported “no” or “do not know,” and one for whom the informant reported no medication (although the participant had reported regular prescribed medication).

##### Group results

Three participants said No to all three awareness of condition items; see Table 3. In the discrepancy items, there was a tendency for participants to overestimate their abilities in all areas in comparison to the informants, apart from the “Impatient” socioemotional item; see Table 4. The items showing the least agreement between the participant and informant, as indicated by the largest mean discrepancy, were the phone and shopping items. For the group overall, “low awareness” discrepancies were more commonly seen for the functional section and the socioemotional section than for the other sections; see Supplementary Figure 2. Discrepancies indicating “high awareness” were infrequently seen, apart from in the radio recall and story recall items, reflecting awareness of memory function and memory performance respectively.

**Table 3.** Responses to awareness of condition items.

Awareness of condition item	Response	*n* (%)
Forgetful	Yes	25 (86.2)
	No	4 (13.8)
Difficulty with concentration	Yes	16 (55.2)
	No	12 (41.4)
	No response	1 (3.4)
Difficulty with your ability to say what you want to say	Yes	17 (58.6)
	No	11 (37.9)
	No response	1 (3.4)
Section total	Yes at least once	26 (89.7)
	No to all responses	3 (10.3)

**Table 4.** Summary of discrepancy item responses and discrepancies.

Self/informant discrepancy item	Participant response median (range); missing	Informant response median (range); missing	Discrepancy mean (SD); missing	Discrepancy median (range)
Mobility	4 (1–4); 1	3 (0–4); 0	0.36 (0.78); 1	0 (−2–2)
Shopping	2 (0–4); 0	0 (0–4); 0	1.03 (1.21); 0	1 (−1–3)
Phone	4 (0–4); 0	3 (0–4); 0	1.03 (1.27); 0	1 (−2–4)
Medication independent	3 (0–4); 2*	2 (0–4); 2*	0.50 (1.33); 3*	0 (−3–3)
Confident	4 (0–4); 0	3 (0–4); 0	0.45 (1.48); 0	0 (−3–4)
Avoid arguments	3 (0–4); 0	2 (0–4); 0	0.41 (1.35); 0	0 (−2–3)
Impatient	3 (0–4); 0	3 (0–4); 0	0.00 (1.31); 0	0 (−2–3)
Comforts others	4 (2–4); 0	3 (0–4); 0	0.79 (1.37); 0	0 (−2–4)
Radio recall	3 (1–4); 0	2 (0–3); 0	0.62 (0.90); 0	1 (−1–2)
Self/performance discrepancy item	Self-rating median (range); missing	Story recall ES median (range); missing	Discrepancy mean (SD); missing	Discrepancy median (range)
Story recall	1 (0–3); 0	1 (0–4); 0	−0.21 (1.61); 0	0 (−4–2)

Note: For all responses, higher scores indicate better ability.

Abbreviation: ES, equivalent score (converted from objective raw score for story recall task).

*Missing as item not applicable.

##### Individual awareness profiles

The three participants who answered No to all three awareness of condition items also showed low awareness in at least one other section. However, there were participants with low awareness in more than one section where this was not reflected in responses to the condition items. Only six participants showed reasonable awareness for all sections. A further five participants showed reasonable awareness in all areas apart from a single negative discrepancy indicating high awareness or underestimation of ability in one area, commonly story recall. There were more positive discrepancies (indicating low awareness) associated with lower cognitive scores. For the story recall item, more participants with higher cognitive scores underestimated their memory performance compared to their objective performance.

#### Interview perspective of participants and informants

Response rates to the structured feedback questions were high, with seven missing responses in total. Five participants and one informant rated any item as either quite difficult or very difficult. There was no apparent demographic pattern for participants who rated items as difficult.

**Figure 2.**
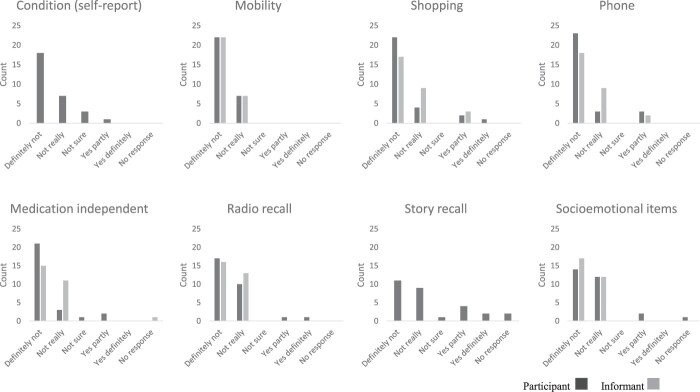
Rating of items as upsetting.

Items were found neither difficult nor upsetting by 14 participants and 25 informants. The most upsetting items for participants were the story recall item and the shopping and phone items; see Figure 2. For the informants, the most upsetting were the shopping and phone items. The reasons given for why items were found upsetting indicate that some participants and informants were upset by questions that reminded them about their diagnosis of dementia, or highlighted current difficulties and changes from past abilities, prompting reflection on losses. No clear pattern was seen in demographics or awareness profile accounting for the ratings.

**Figure 3.**
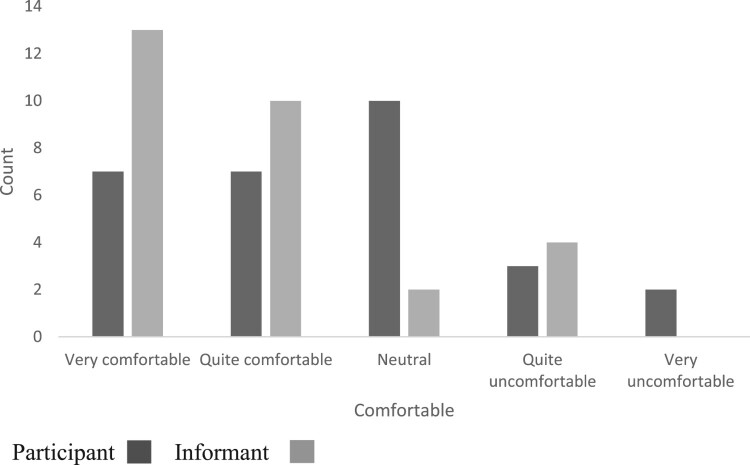
Overall interview feedback.

In contrast to item feedback, the overall interview feedback showed fewer negative ratings, with only five participants rating the experience as quite uncomfortable or very uncomfortable, and four informants rating it as quite uncomfortable; see Figure 3. Three of the participants had shown reasonable awareness in most areas but had underestimated their memory performance.

#### Quantitative content analyses of feedback comments

There were 24 participants and 22 informants who gave additional comments about the overall interview. The hypotheses tested and coding have been summarized in Supplementary Table 2. Most people appeared to find the interview acceptable and easy to understand, with no indication that it was any more taxing than previous clinical experiences. Acknowledgement was made that assessments can be challenging, and highlighting daily difficulties can be upsetting, but acceptable. For the informant, providing responses away from the participant was important, and being able to clarify responses with the interviewer was helpful.

Following the story recall task, five participants rated it as “challenging but tolerable,” three as a “neutral” experience, and one as “unpleasant.” None rated it as enjoyable and 20 opted to provide other comments in their own words. A summary of the quantitative content analysis of their comments is shown in Supplementary Table 3. Two of the participants had given overall interview ratings as quite uncomfortable or very uncomfortable. One of these participants was unhappy with the story recall task and felt it was not needed. The other participant described difficulty with the task which highlighted problems in an unhelpful way. They had both underestimated their performance on the story recall task and had reasonable awareness in other sections.

Overall, the story recall item was more demanding than the other items, particularly for participants with higher awareness of their memory performance. For two participants this may have influenced their overall discomfort with the interview, but for the majority, the story recall item appears to have been acceptable.

#### Field notes and researcher observations

These noted the importance of clear instructions and support from the interviewer when asking the questions, particularly for the mobility and medication items. Remote administration was uncomplicated. Most of the participants found the colour response chart helpful, and it was considered essential by one dyad.

### Revisions and finalization of the new tool

Following the analysis of the pilot study results, areas for review were listed; see Supplementary Table 4. These address issues of feasibility, acceptability, and clinical utility of the awareness interview, and were discussed in separate online meetings with the research team, the PPI group, and the clinical advisory group.

#### Preliminary discussions

The wording in some items and the story recall test were reviewed considering concerns recorded in the field notes, with additional instructions for the mobility and medication items. The reverse-scored socioemotional item “I am impatient” was found potentially confusing and did not contribute significantly to the overall awareness profile, so was removed.

#### Clinical advisory group discussion

The format of the awareness interview was reviewed, with suggestions for easier use by clinicians. It was thought that the length of the assessment might be a barrier to routine use, but feasible for tailored community assessments. Reducing the interview by one item as discussed will reduce the time required to approximately 10 min. Areas were identified where examining awareness could support assessments by community health and social care teams; see Supplementary Text for details.

#### PPI group discussion

Discussion included the challenges of remote assessments for some people with dementia, the importance of simple language in the instructions, and the conflicting experiences of carers acting as informants. The latter could sometimes invoke feelings of disloyalty but was balanced by a positive sense of wanting to help the other person, and the opportunity to raise difficult issues. There was a concern that the dramatic story used in the story recall test could have a negative impact on the person with dementia. On review, story recall tests commonly report a dramatic event (Lezak et al., 2012), which can enhance recall (Bradley et al., 1992), but older people are more likely to remember positive information (Gorenc-Mahmutaj et al., 2015) and maintain emotional well-being in the face of stressful situations (Carstensen et al., 2020). As there were limited options for maintaining the standardized scoring, the story was left largely unchanged.

#### Scoring and terminology

The scoring for the interview results in awareness bands for each section and is not designed to give an overall score. This is in keeping with the evidence that awareness manifests differently across different objects and different assessment methods (Clare, Marková, et al., 2011; Marková et al., 2014). For participants who do not have someone available to act as an informant, it would be possible to use a reduced set of items to assess awareness, i.e., the three self-reported condition items and the memory performance item could provide some information in restricted circumstances. However, the full interview is recommended where there is an available informant.

The terminology used for the awareness bands refers to low, reasonable, and high awareness. These terms were chosen for simplicity for clinical use but merit some explanation. Low awareness is indicated by a positive discrepancy i.e., where the person with dementia overestimates ability in comparison to the informant rating or objective memory performance. Lack of endorsement of any of the three common dementia symptoms is also considered to reflect low awareness. In contrast, negative discrepancies i.e., underestimation of ability in comparison with informant rating or objective memory performance, are described as high awareness. This can be better understood as heightened awareness, i.e., the person has a heightened awareness of difficulties, which might not necessarily equate to a more accurate appraisal of abilities. The awareness bands produce a profile of awareness across different domains, reflected in the name of the tool.

The current version of the HAPI awaits validation and can be obtained from the corresponding author on request.

## Discussion

Using research recommendations, a new clinical tool was developed for assessing awareness in people with dementia, to support person-centred care. The tool was designed to inform clinical interactions to facilitate tailored support for people with mild-to-moderate dementia and carers. It assesses awareness over a range of domains and includes awareness of a physical condition via the mobility item, in recognition of the challenge of multimorbidity in people with dementia. It employs a range of methods, assessing awareness at an evaluative level using the discrepancy between self-ratings and informant ratings as an index of awareness, as well as awareness of performance on a memory task and subjective expression of awareness of having dementia symptoms. The tool, designed as a structured clinical interview, was tested in a small pilot study and found suitable for people with MoCA-5 min scores ranging of 9.5 or above. A range of individual awareness profiles was formed. The overall interview was generally found easy to understand, and acceptable by participants and informants. It was not suitable for people with MoCA-5 min scores ≤3, but further investigation is required regarding suitability when the MoCA-5 min score is between 3 and 9.5. Administration by telephone or videoconference was feasible. Areas were identified where awareness profiling could potentially enhance health and social care assessments in the community.

In comparison, there are two short-form awareness measures brief enough for clinical use, which rely on informant ratings (Dourado et al., 2019; Turró-Garriga et al., 2014), and the benefits and limitations of using these for clinical purposes have been discussed in detail previously (Alexander, Martyr, Savage, et al., 2021). The primary difference between these measures and the HAPI is that the latter was designed specifically for clinical use and to assess specific area(s) of need in people with dementia. The HAPI does not solely rely on the accuracy of ratings to assess awareness as it includes a measure of memory ability. This means that unlike earlier measures it does not rely solely on the accuracy of perceived abilities (Dourado et al., 2019; Turró-Garriga et al., 2014). This memory component can be used to assess the accuracy of both the person with dementia and the carer in relation to their appraisal of memory ability. While not directly related to other objects of awareness included in the HAPI, where there is good concordance between memory ratings and memory ability this could increase the confidence in the accuracy of the other ratings in the HAPI. Informant ratings are important in research and in clinical assessments (e.g., Jorm, 2004) but limitations are recognized (Conde-Sala et al., 2013; Hanson & Clarke, 2013; Martyr & Clare, 2018). Items comparing objective performance with self-rating are valuable as they reveal a different aspect of awareness (Clare et al., 2013) but are infrequently included in multi-domain measures (Alexander, Martyr, Savage, et al., 2021).

An important consideration is whether assessment causes distress. Short-term distress from cognitive testing can be related to awareness of cognitive difficulties rather than the actual test performance (Lai et al., 2008). Participants who were particularly unhappy with the story recall task showed high awareness of their memory performance and reasonable awareness in other areas. Assessing awareness could help identify the people most likely to experience distress during cognitive testing, and in everyday tasks, and who might benefit from supportive strategies or interventions (Clare et al., 2019; Clare et al., 2004; Lai et al., 2008). In practice, clinicians may be more likely to use the HAPI where there are existing, clinical concerns about low awareness. Other participants described the task as challenging but necessary, and not without benefit. This attitude is recognized in clinical assessments, where testing is often not enjoyed, but acceptable, particularly when the intentions are understood, and the assessment is perceived as thorough (Lee et al., 2018).

### Strengths and limitations

Strengths: Tool development was led by evidence from awareness research, producing a multidimensional assessment. The novel inclusion of an item assessing awareness of mobility adds to the clinical utility. Falls and falling are a common problem in older people, and while not all people with dementia experience falls, awareness of mobility issues is useful to assess the potential for fall risk. As there was little difference between self- and informant ratings for mobility, any discrepancy, especially where the person with dementia underestimates the risk of falls compared to their carer, could be an indicator of poorer awareness of walking difficulty or fall risk. The inclusion of an objective measure of memory was a strength as this means the HAPI does not solely rely on ratings and the discrepancy between the person with dementia and their carer to assess awareness. Including the memory assessment also means that where there is no available carer an abridged version of the HAPI can still be administered and an assessment of awareness can be made. The involvement of experienced clinicians from primary and secondary care services was valuable, allowing useful discussion and generation of ideas for clinical use of the interview. The contribution of the PPI group was essential in developing and revising the interview, and ensuring acceptability was satisfactory for people with lived experience of dementia.

Limitations: The tool was intentionally brief, which restricted the range of items included. The time required to administer the interview may still be too long for some settings. The awareness of condition items may be less applicable to people with rarer dementia subtypes, although this was not apparent when examined in the IDEAL dataset (Alexander, Martyr, Gamble, et al., 2021). There was little cultural diversity among the participants and the informants. Socioeconomic status was not assessed. Expressed awareness can differ between cultural groups (Mograbi, Ferri, et al., 2012) and socioeconomic groups may influence awareness of condition and diagnosis (Alexander, Martyr, Gamble, et al., 2021). These factors may also influence the suitability and acceptability of the items for people from different backgrounds. A larger validation study should therefore include participants from a range of social and cultural backgrounds.

Using the MoCA-5 min to corroborate the participant’s self-defined appraisal of having mild or moderate dementia was a limitation, as screening measures are not comprehensive assessments of cognition. However, as the HAPI is primarily designed for clinical use, this limitation is unlikely to affect subsequent uses of the measure as comprehensive assessments of cognition are more likely to occur in these settings. Including the MoCA-5 min at the end of the study was a limitation as it meant that two people had to be excluded after they completed the HAPI due to their difficulty completing the interview with a considerable number of non-responses. These two people subsequently scored well below the MoCA-5 min inclusion score for the study (scoring 2 and 3 on the MoCA-5 min). It was necessary to include the MoCA-5 min at the end of the study rather than as a screening tool for inclusion before the interview as the MoCA-5 min includes a memory assessment and performance on this measure could have influenced ratings on the memory component of the HAPI. Screening for inclusion in the study on a different day could have prevented this but this could also have increased the potential for burden on the participants. This may be less applicable in larger studies or in clinical settings where assessments might happen over several weeks. Inviting the carer to remain in the room during the HAPI was a limitation as this may have influenced how the person with dementia responded to the awareness questions. The carer was invited to remain in the room so the researcher could be alerted if there was any cause for concern such as technical problems or distress caused; however, ideally, the HAPI should be completed separately by both the person with dementia and the carer to avoid responses by one member of the dyad influencing the ratings of the other.

The pilot study took place during the coronavirus pandemic. For the participants, usual activities were changed or restricted due to lockdown rules or self-isolation guidance, which may have made it more difficult to describe usual functioning. This was for many people an early experience of being assessed remotely, which may have increased the reported difficulty with the HAPI, though few participants expressed any difficulty completing the interview. The researcher was conducting the interviews remotely from a home environment with unlimited time to interview each participant in a non-pressurized environment, unlike a typical clinical appointment; this may have altered the perception of how threatening or acceptable the questions were. The interview has not yet been administered face-to-face, but this is expected to be no more difficult than remote administration, especially as many of the items were adapted from or similar to items from measures that have previously been extensively administered face-to-face.

## Conclusions

The HAPI offers a brief structured approach to assessing awareness in clinical settings, without reliance on clinical observation alone, or lengthy research measures. Awareness profiling could be used to guide person-centred care in various healthcare and social care settings. As a pragmatic tool, the HAPI could be used to gather information, start important conversations, document the current position concerning awareness, map changes in awareness, and provide a shared language to describe awareness issues between members of a multidisciplinary team. The awareness profile could facilitate discussions at the time of diagnosis and afterwards. The information afforded could highlight where people with dementia and/or carers need additional help either with psychological support, practical help, and/or supervision. This may guide information provision, support, and signposting/onward referrals to specialist centres, where required. Where awareness of difficulties varies within the dyad, negotiation of how to proceed could be facilitated in dyadic work. A dyadic intervention approach has been valuable elsewhere for selected dyads (Whitlatch et al., 2006) and could help develop mutual understanding and agreement within the dyad on care issues. Identifying areas where perspectives differ between the person with dementia and the carer could enable supportive interventions. With this different approach, the HAPI may be a useful addition to available tools for assessing people with dementia.

## Supplementary Material

Supplementary Material HAPI.docx

## Data Availability

Requests for access to the data should be made to the first author at REACH: the Centre for Research in Ageing and Cognitive Health. The current version of the Healthcare Awareness Profile Interview is awaiting validation and is included in the first author’s PhD thesis. Details are available on request.
