# Supplementary material for: Healthcare Awareness Profile Interview: Development of a new evidence-based brief clinical tool to assess awareness in people with dementia
Source: Neuropsychol Rehabil. 2024 Apr 17;35(2):292–315. doi: 10.1080/09602011.2024.2337152 (PMC11854051; doi:10.1080/09602011.2024.2337152)
Supplement: Supplementary Material HAPI.docx [file PNRH_A_2337152_SM3620.docx]

The Healthcare Awareness Profile Interview: Development of a new evidence-based brief clinical tool to assess awareness in people with dementia

# SUPPLEMENTARY MATERIAL

## List of Supplementary Material

- Supplementary Text
- Supplementary Table 1. Items included in pilot version of awareness interview
- Supplementary Table 2. Summary of content analysis for participant and informant overall interview comments
- Supplementary Table 3. Summary of content analysis for other comments about the story recall task
- Supplementary Table 4. Areas for review in discussion groups
- Supplementary Figure 1. Procedure for pilot study interviews
- Supplementary Figure 2. Section discrepancies indicating score bands

## Supplementary Text

### **Analyses and Proposed Scoring**

Item responses were analysed to show the range and frequency of responses, and frequency of non-responses. For the three self-reported awareness of condition items, a total score was calculated for the number of items endorsed. This was converted to a binary score to indicate whether the participant said No to all three items, i.e., did not acknowledge any of these three common dementia symptoms, considered to indicate ‘low awareness’, or said Yes to at least one item, considered to indicate ‘reasonable awareness’.

For applicable items, a discrepancy score was calculated by subtracting the value for informant rating from the participant rating. For the story recall item, the discrepancy was calculated by subtracting the story recall equivalent score from the participant self-rating of performance. Section discrepancies were formed by adding the discrepancies for shopping, phone, and medication independent items to create the functional section discrepancy, and the four socioemotional item discrepancies were added to form the socioemotional section discrepancy.

Discrepancies within the range ±1 for each item are considered unremarkable, or ‘reasonable awareness’. Discrepancies larger than ±1 per item are considered to indicate altered awareness, i.e., ‘low awareness’ if the discrepancy is positive (indicating the person with dementia overestimated ability compared to the informant rating), and ‘high awareness’ if the discrepancy is negative (indicating the person with dementia underestimated ability compared to the informant rating). The functional section score combines the discrepancy for three items; therefore, the score band for reasonable awareness is between -3 and +3. The socioemotional section score in the pilot version combines the discrepancy for four items; therefore, the score band for reasonable awareness is between -4 and +4.

Where discrepancies are minimal or zero, i.e., self-rating of ability is in line with informant ratings or objective performance, and when at least one common symptom of dementia is acknowledged in the awareness of condition items, there is no evident difficulty with awareness. For each section, this middle band is described as ‘reasonable’ awareness.

### **Pilot Study Participants: Details of Exclusion Criteria**

Exclusion criteria were difficulty managing a telephone call or videoconference of around 45 minutes, or inability to provide consent to take part. Recruitment targeted people with mild-to-moderate dementia, defined here as having a score of ≥11 on the Montreal Cognitive Assessment Five-minute protocol (MoCA-5 min; Wong et al., 2018; Wong et al., 2015), which according to Wong et al (2018) is equivalent to a score of ≥18 on the Mini-Mental State Examination (MMSE; Folstein et al., 1975).

The necessary order of administration of measures in the study meant that the cognitive test could only be administered at the end of the interview. Therefore, the MoCA-5 min score was not used as a criterion for inclusion. Potential participants self-selected based on perceiving themselves as having mild-to-moderate dementia. Data from participants with cognitive scores below the target were included in the analysis if participants were able to manage the interview satisfactorily with responses documented to most or all items.

### **Pilot Study Participants: Demographic Details**

Demographic information recorded from participants and informants included age in years (categorised afterwards into age groups <65y, 65-69y, 70-74y, 75-79y, and 80+y), sex, ethnic group (*white, Asian/Asian British, black/African/Caribbean/black British, mixed/multiple ethnic groups, other ethnic group*), age on leaving school, highest qualification achieved (no formal qualifications, age 16 school leaving certificate, age 18 school leaving certificate, university qualification). In addition, the informant was asked to categorise their relationship with the participant (spouse/partner, child, sibling, other relation, friend) and how long they had known each other. Information about the dementia subtype and length of time since diagnosis was obtained either from the JDR online portal or research team contact lists beforehand; for two participants this information was volunteered by the informant. Length of time since diagnosis was grouped into <1y, 1-2y, 3-5y, 6+y.

### **Pilot Study: Feedback Questions**

Feedback questions for the participant and informant were inserted between items and asked if the questions were easy or difficult to understand, rated on a five-point scale from ‘very easy’ to ‘very difficult’. The participant was also asked whether the questions were upsetting in any way, rated on a five-point scale from ‘no definitely not’ to ‘yes definitely’, with an invitation to say which parts were upsetting where applicable. After the story recall item, the participant was asked how they had found the task, with response options ranging from enjoyable to unpleasant, and an option to provide other comments. At the end of the interview or questionnaire, the participants and informants were asked to rate how comfortable they would feel if asked to do this assessment in clinic, rated on a five-point scale from very comfortable to very uncomfortable. They were also invited to give any other comments about their experience of taking part.

### **Results: Item Selection for the Pilot Version of the Awareness Tool**

#### Awareness of Memory Function and Performance.

The Memory Awareness Rating Scale (MARS; Clare et al., 2002) memory performance item for story immediate recall had high item-total correlation, with good face validity for everyday memory challenges. It was judged to be feasible for administering over the telephone or videoconference and would fit into a brief assessment. The story used with the MARS is part of the Rivermead Behavioural Memory Test (Wilson et al., 2007) and is therefore subject to copyright. An alternative story that has been used in a similar way is the Babcock story recall test (Lezak et al., 2012). This has been shown to have good construct validity for memory recall (Horner et al., 2002) and normative data are available (Carlesimo et al., 2002). The use of standardised scores has been demonstrated in several studies (Di Carlo et al., 2007; Maggi et al., 1994; Solfrizzi et al., 2004; Solfrizzi et al., 2015). Calculation of a discrepancy between the self-evaluation and objective performance can be used as an indication of awareness of memory performance (Morris et al., 2016).

In the HAPI, the story recall task is rated by the interviewer using a template with points scored for themes recalled. Raw scores are expressed as standardised profile scores using cut-offs and converted to an equivalent score on a scale of 0 to 4, allowing the discrepancy with self-rating of performance to be calculated.

Corresponding to the memory performance item, the selected item for awareness of memory function in the MARS is about hearing a news story and being able to repeat it to someone else afterwards. This item uses the discrepancy between self-rating and informant rating as an indication of awareness. The inclusion of items assessing awareness of both memory performance and function is supported by research demonstrating the difference between these levels of awareness (Clare et al., 2013).

#### Awareness of Functional Ability.

From the Functional Activities Questionnaire (FAQ; Pfeffer et al., 1982), the shopping item enquiring about being able to shop alone had the highest item-total correlation in data from the Memory Impairment and Dementia Awareness Study (MIDAS; Clare et al., 2012), showing no sex bias. The modified-FAQ (Martyr et al., 2012) telephone item about being able to use a telephone independently was considered relevant for care assessments, particularly for people living alone, and showed a moderate item-total correlation in the MIDAS data. It was therefore adapted for use in the new tool and known as the ‘phone’ item. A new question was formulated about prescribed medication management that could be used to calculate a discrepancy score between self- and informant ratings. While most people with dementia take regular prescribed medication this is not universal, therefore this item was only administered if the participant said that they do take regular prescribed medication. In cases where they do not know or report no regular prescribed medication this item is not included in the scoring.

#### Awareness of Socioemotional Functioning.

Using item-total correlations from the Socio-Emotional Questionnaire (SEQ; Bramham et al., 2009) responses in the MIDAS data (Nelis et al., 2011), one item was selected to represent each of the three identified SEQ factors (Nelis et al., 2011). The selected items were ‘when others are sad I comfort them’, ‘I am confident meeting new people’, and ‘I avoid arguments’. An additional item requiring reversed scoring was included to see if negatively phrased items would be a useful contribution, ‘I am impatient with other people’. This reverse-scored item was subsequently removed from the HAPI after the pilot study.

#### Awareness of Condition.

Using data from the IDEAL cohort (Clare et al., 2014) three questions were selected from the screening checklist for awareness of condition in the Representations and Adjustment to Dementia Index (RADIX; Quinn et al., 2018). Exploration of pattern responses in the full nine-item checklist (see Alexander et al, 2022) identified the items that were best able to identify people with low awareness of their condition.

#### Awareness of Physical Condition: Mobility.

Exploring the EQ-5D-3L (The EuroQol Group, 1990) mobility data in the IDEAL baseline dataset, the discrepancy between participant and informant responses indicated that the participant overestimated their mobility relative to the informant rating. Subsequently, a new mobility awareness item was developed using a five-point response scale in line with the other selected items included in the HAPI.

### **Clinical Advisory Group Discussion About Clinical Utility of Awareness Assessment**

For people with dementia living at home, awareness assessment could usefully be incorporated into a number of community-based reviews, for example in the UK, by reablement/rehabilitation teams when supporting individuals to maintain independence after hospital discharge (National Institute for Health and Care Excellence, 2017). The HAPI could be included in the holistic assessments of people who have dementia with other co-morbidities, by teams such as community frailty services (Moody et al., 2017), to optimise care and support and promote admission avoidance. The HAPI could help provision of tailored care by dementia support professionals, for instance, Admiral Nurses (Aldridge & Harrison Dening, 2019; Dementia UK, 2021) and other organisations such as Alzheimer’s Society (Alzheimer's Society, 2020). Social care assessments, for example for Continuing Health Care needs (Brooker et al., 2015), might benefit from recognising difficulties with awareness when assessing people with dementia. Dementia reviews carried out in primary care (National Institute for Health and Care Excellence, 2018; Wheatley et al., 2022) could be more effective and person-centred if awareness concerns were identified and documented in a standardised way (NHS Digital, 2020).

## Supplementary Tables and Figures

Supplementary Table 1. Items included in the pilot version of the awareness tool

Participant is the person with dementia; informant is the carer

| **Awareness domain** | **Origin of item** | **Rationale for inclusion** | **Type of item** | **Scoring** |
| --- | --- | --- | --- | --- |
| Awareness of condition | RADIX Screening Questions | - Items selected from item response analyses to RADIX screening questions in version 4 of the IDEAL T1 data, choosing three items that were able to distinguish the group of people with dementia who endorsed none of the nine screening questions (i.e., had lower awareness of condition) from those who endorsed at least one question. - Items were excluded if too general (‘Are you different in some way?’) or too similar to other included items (‘Difficulty with remembering’ similar to ‘Have been forgetful’, ‘difficulty in thinking’ similar to ‘difficulty with concentration’). - The three selected items showed effective identification of a low awareness group when compared to using the full nine-item RADIX checklist. | Self-rating by participant. | Each item scored 0 for Yes and 1 for No. A total score of 3 (responded No to all 3 questions) indicates low awareness. A score of 0-2 (endorsement of at least one item) indicates some (reasonable) awareness. |
| Awareness of mobility | Developed item after reviewing other scales and examining responses from EQ-5D-3L question on mobility | - Discrepancy between responses by participant and informant responses to EQ-5D-3L mobility question in version 4 of the IDEAL T1 data was significant predictor of awareness of condition group. - Designed new item with new five-response scale. | Discrepancy between participant rating of own mobility and informant rating of participant mobility. | Rated on five-point Likert scale (0 to 4) leading to discrepancy of -4 to +4. Discrepancy is participant rating minus informant rating. More positive discrepancy indicates that participant over-estimates mobility compared to informant. |
| Awareness of functional ability | Shopping item and phone item: adapted from FAQ as used in MIDAS.  Medication item: modified from OARS-iADL and CSADL items. | - Shopping item: Highest item-total correlation (r=.644) for FAQ in MIDAS data. - Phone item considered for reasons of face validity, useful area to assess. However lower in ranking of item-total correlations (MIDAS r=.488, ranked ninth of 11 FAQ items). - Medication item derived from similar iADL items in other validated measures. Face validity, useful item for healthcare measure, but unproven reliability or validity as an awareness item. | Discrepancy between participant self-rating and informant rating. | Rated on five-point Likert scale (0 to 4) leading to discrepancy score -4 to + 4 for each item. These three items can be summed for a functional awareness score. Discrepancy is participant rating minus informant rating. More positive discrepancy indicates participant overrates ability. |
| Awareness of memory function | MARS- MFS | - High item-total correlation (r=.727) for MFS story immediate recall item in MIDAS data (second to story delayed recall item) - Corresponding memory performance item (MARS-MPS) testing immediate recall of short story is feasible by remote assessment by telephone or videoconferencing - Feasible in brief measure (story delayed item would require longer time for assessment). | Discrepancy between participant rating and informant rating. | Rated on five-point Likert scale (0 to 4) for responses, leading to discrepancy score of -4 to +4.  Discrepancy is participant rating minus informant rating.  More positive discrepancy indicates participant overestimates ability compared to informant. |
| Awareness of memory performance | MARS-MPS with short story immediate recall item from modified Babcock Story recall test. | - High item-total correlation (r=.469) for MPS story immediate recall item in MIDAS data (second to story delayed recall item). - Feasible by remote assessment - Manageable in a brief assessment. | Discrepancy between objective performance on recall task and self-evaluation after the task. | Five-point Likert scale (0 to 4) for self-evaluation. Objective task rated by interviewer using template with points scored for themes recalled. Raw scores expressed as standardised profile score using cut-offs, then converted to score 0 to 4 so discrepancy with self-rating can be calculated.  Discrepancy is self-rating minus performance converted score. More positive discrepancy indicates that participant overestimates ability compared to actual performance. |
| Awareness of socioemotional functioning | SEQ | - Items selected from MIDAS SEQ data with highest item-total correlations for each of the three factors validated in dementia, social relationships (SR), emotional recognition and empathy (ERE), and prosocial behaviour (PB). - ERE item ‘when others are sad I comfort them’ item-total correlation r=.621 - SR item with highest item-total correlation was ‘I am sociable’. However large majority of discrepancy values were zero with narrow distribution. Second highest correlation is ‘I am confident meeting new people’, which also has face validity so selected instead. - PB factor items generally had lower correlation with total score. Highest was ‘I avoid arguments’, correlation r=.301. Others showed poor correlation (<.3). - PB item ‘I am impatient with other people’ came a close second with item-total correlation r=.295. Included in pilot as other items all worded positively and may encourage unidirectional responding. | Discrepancy between participant self-rating and informant rating. | Rated on five-point Likert scale (0 to 4) leading to discrepancy score -4 to + 4 for each item. These can be summed for a socioemotional awareness score.  ‘I am impatient’ is reverse scored. Discrepancy is participant rating minus informant rating. More positive discrepancy indicates that participant overestimates socioemotional functioning compared to informant. |

Abbreviations. RADIX Representations and Adjustment to Dementia Index; IDEAL Improving the experience of Dementia and Enhancing Active Life; FAQ Functional Activities Questionnaire; MIDAS Memory Impairment and Dementia Awareness Study; CSADL Cleveland Scale for Activities of Daily Living (Patterson et al., 1992); OARS-iADL Older Americans Resources and Services iADL (Fillenbaum, 1988); iADL instrumental activities of daily living; MARS-MFS Memory Awareness Rating Scale-Memory Function Scale; MARS-MPS Memory Awareness Rating Scale-Memory Performance Scale; SEQ Socio-Emotional Questionnaire.

Supplementary Table 2. Summary of content analysis for participant and informant overall interview comments*

|  | Participant (n=24) | Informant (n=22) |
| --- | --- | --- |
| Hypothesis 1: Most people found the awareness interview acceptable and easy to understand |  |  |
| Happy with interview | 9 | 3 |
| Interview is straightforward | 4 | 4 |
| Separate rooms important | - | 3 |
| Measure suggestions | - | 7 |
| Measure concerns | - | 3 |
| Hypothesis 2: The awareness interview was not more taxing than experiences in the dementia diagnostic process. |  |  |
| Assessments can be challenging | 4 | 2 |
| Assessments hold no fear | 2 | - |
| Research Question: What other reasons did people give in support of their overall reaction to the interview? |  |  |
| Interview promotes reflection | 13 | 5 |
| Research interview helps others | 3 | 1 |

*Hypotheses were developed drawing on the expertise of researchers and clinicians in the research team, earlier feedback from a patient and public involvement group about the interview, research on the acceptability of cognitive assessments (Lai et al., 2008; Lee et al., 2018), and the prior structured responses to feedback questions in the pilot study. Using the comments made by participants and informants as data, coding labels were developed from an overview of the data, relevant to the hypotheses. The initial coding was conducted by the first author and reliability was tested by comparing data coding with the last author.

Supplementary Table 3 Summary of content analysis for other comments about the story recall task

| Hypothesis: People with dementia find cognitive assessments are challenging, but acceptable and necessary  Research question: In their own words, how did the participants find the memory/story recall task? | Participants (n=20) |
| --- | --- |
| Challenging? |  |
| Challenge difficult | 9 |
| Challenge OK | 2 |
| Emotional response |  |
| Happy with task | 10 |
| Unhappy with task | 7 |
| Is it necessary? |  |
| Wanted/necessary | 3 |
| Unwanted/unnecessary | 2 |
| Highlights difficulties | 7 |
| Next time | 1 |

*Hypotheses were developed as described above. Initial coding by the first author was reviewed by the last author.

Supplementary Table 4. Areas for review in discussion groups

| Category | Issue | Origin |
| --- | --- | --- |
| *Feasibility* | Importance of reverse wording and/or item reduction for the socioemotional section | Tool development |
|  | Review of instructions for the medication and mobility questions | Field notes |
|  | Review of wording for story recall news story | Field notes |
|  | Materials formatted for clinical interview | Future use |
|  | Remote or face-to-face assessments in dementia care | Future use |
|  | Timing/length of the interview | Pilot study results and future use |
| *Acceptability* | Remote assessments | Field notes |
|  | Managing potentially upsetting items; story recall task | Pilot study feedback |
|  | Informant role | Pilot study feedback |
| *Clinical utility* | Awareness profile and awareness bands | Pilot study results |
|  | Who is the interview for? | Future use |
|  | When would it be used? | Future use |
|  | Proposed scoring | Tool development |


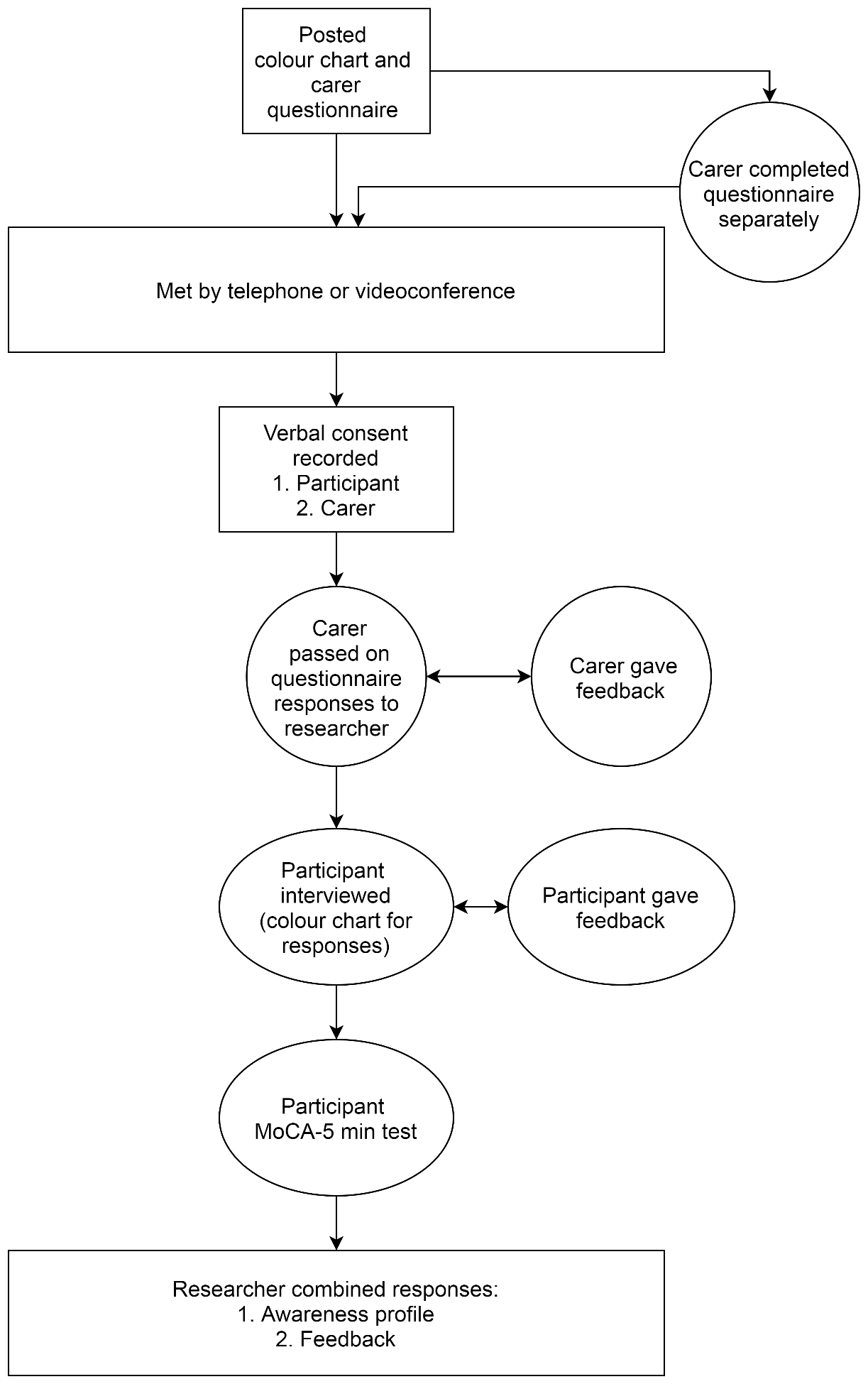


Supplementary Figure 1. Procedure for pilot study interviews

Abbreviation. MoCA-5 min Montreal Cognitive Assessment Five-minute protocol

| Functional section n=26 | Socioemotional section n=29 | Mobility n=28 |
| --- | --- | --- |
|  |  |  |
| Radio recall (memory function) n=29 | Story recall (memory performance) n=29 |  |
|  |  | \|  \| Reasonable awareness  High awareness  Low awareness \| \| --- \| --- \| |

Functional section combines discrepancies for three items: shopping, phone, and medication independent. Functional section discrepancy is not shown for three participants, for whom the medication item was not scored. Socioemotional section combines discrepancies for four items: confident, avoids arguments, impatient, and comforts sad. Mobility item had missing data for one participant.

Supplementary Figure 2. Section discrepancies indicating score band

# REFERENCES

Aldridge, Z., & Harrison Dening, K. (2019). Admiral nursing in primary care: peri and post-diagnostic support for families affected by dementia within the UK primary care network model. *OBM Geriatrics*, *3*(4), 081. <https://doi.org/10.21926/obm.geriatr.1904081>

Alzheimer's Society. (2020). *The Dementia Guide: Living well after your diagnosis*. <https://www.alzheimers.org.uk/sites/default/files/2020-03/the_dementia_guide_872.pdf>

Bramham, J., Morris, R. G., Hornak, J., Bullock, P., & Polkey, C. E. (2009). Social and emotional functioning following bilateral and unilateral neurosurgical prefrontal cortex lesions. *Journal of Neuropsychology*, *3*(1), 125-143. <https://doi.org/10.1348/174866408X293994>

Brooker, D., Milosevic, S., & Yemm, H. (2015). *Guidance for NHS continuing healthcare assessors: Evaluating emotional and psychological needs for people in the later stages of dementia*. <https://www.worc.ac.uk/documents/2015-Guidance-for-NHS-continuing-healthcare-assessors.pdf>

Carlesimo, G. A., Buccione, I., Fadda, L., Graceffa, A., Mauri, M., Lorusso, S., Bevilacqua, G., & Caltagirone, C. (2002). Normative data of two memory tasks: Short-Story recall and Rey's Figure. *Nuova Rivista di Neurologia*, *12*(1), 1-13.

Clare, L., Nelis, S. M., Martyr, A., Roberts, J. L., Whitaker, C. J., Marková, I. S., Roth, I., Woods, R. T., & Morris, R. G. (2012). The influence of psychological, social and contextual factors on the expression and measurement of awareness in early-stage dementia: testing a biopsychosocial model. *International Journal of Geriatric Psychiatry*, *27*(2), 167-177. <https://doi.org/10.1002/gps.2705>

Clare, L., Nelis, S. M., Quinn, C., Martyr, A., Henderson, C., Hindle, J. V., Jones, I. R., Jones, R. W., Knapp, M., Kopelman, M. D., Morris, R. G., Pickett, J. A., Rusted, J. M., Savitch, N. M., Thom, J. M., & Victor, C. R. (2014). Improving the experience of Dementia and Enhancing Active Life - living well with dementia: study protocol for the IDEAL study. *Health and Quality of Life Outcomes*, *12*(1), 164. <https://doi.org/10.1186/s12955-014-0164-6>

Clare, L., Whitaker, C. J., Roberts, J. L., Nelis, S. M., Martyr, A., Marková, I. S., Roth, I., Woods, R. T., & Morris, R. G. (2013). Memory awareness profiles differentiate mild cognitive impairment from early-stage dementia: Evidence from assessments of performance monitoring and evaluative judgement. *Dementia and Geriatric Cognitive Disorders*, *35*(5-6), 266-279. <https://doi.org/10.1159/000346735>

Clare, L., Wilson, B. A., Carter, G., Roth, I., & Hodges, J. R. (2002). Assessing awareness in early-stage Alzheimer's disease: development and piloting of the Memory Awareness Rating Scale. *Neuropsychological Rehabilitation*, *12*(4), 341-362. <https://doi.org/10.1080/09602010244000129>

Dementia UK. (2021). *Dementia specialist Admiral Nurses*. <https://www.dementiauk.org/wp-content/uploads/2021/06/DUKIL05_AdmiralNurse_Online.pdf>

Di Carlo, A., Lamassa, M., Baldereschi, M., Inzitari, M., Scafato, E., Farchi, G., & Inzitari, D. (2007). CIND and MCI in the Italian elderly: frequency, vascular risk factors, progression to dementia. *Neurology*, *68*(22), 1909-1916. <https://doi.org/10.1212/01.wnl.0000263132.99055.0d>

Fillenbaum, G. G. (1988). *Multidimensional functional assessment of older adults: The Duke Older Americans Resources and Services procedures*. Lawrence Erlbaum Associates, Inc. <https://doi.org/10.4324/9780203771563>

Folstein, M. F., Folstein, S. E., & McHugh, P. R. (1975). “Mini-mental state”: a practical method for grading the cognitive state of patients for the clinician. *Journal of Psychiatric Research*, *12*(3), 189-198. <https://doi.org/10.1016/0022-3956(75)90026-6>

Horner, M. D., Teichner, G., Kortte, K. B., & Harvey, R. T. (2002). Construct validity of the Babcock Story Recall Test. *Applied Neuropsychology*, *9*(2), 114-116. <https://doi.org/10.1207/S15324826AN0902_7>

Lai, J. M., Hawkins, K. A., Gross, C. P., & Karlawish, J. H. (2008). Self-reported distress after cognitive testing in patients with Alzheimer's disease. *The Journals of Gerontology. Series A, Biological Sciences and Medical Sciences*, *63*(8), 855-859. <https://doi.org/10.1093/gerona/63.8.855>

Lee, L., Slonim, K., Hillier, L. M., Lu, S. K., & Lee, J. (2018). Persons with dementia and care partners’ perspectives on memory clinics in primary care. *Neurodegenerative Disease Management*, *8*(6), 385-397. <https://doi.org/10.2217/nmt-2018-0024>

Lezak, M. D., Howieson, D. B., Bigler, E. D., & Tranel, D. (2012). *Neuropsychological Assessment* (5th ed.). Oxford University Press.

Maggi, S., Zucchetto, M., Grigoletto, F., Baldereschi, M., Candelise, L., Scarpini, E., Scarlato, G., Amaducci, L., & Group, I. (1994). The Italian Longitudinal Study on Aging (ILSA): design and methods. *Aging Clinical and Experimental Research*, *6*(6), 464-473. <https://doi.org/10.1007/BF03324279>

Martyr, A., Clare, L., Nelis, S. M., Marková, I. S., Roth, I., Woods, R. T., Whitaker, C. J., & Morris, R. G. (2012). Verbal fluency and awareness of functional deficits in early-stage dementia. *The Clinical Neuropsychologist*, *26*(3), 501-519. <https://doi.org/10.1080/13854046.2012.665482>

Moody, D., Lyndon, H., & Stevens, G. (2017). *Toolkit for general practice in supporting older people living with frailty*. NHS England.

Morris, R. G., Nelis, S. M., Martyr, A., Markova, I., Roth, I., Woods, R. T., Whitaker, C. J., & Clare, L. (2016). Awareness of memory task impairment versus everyday memory difficulties in dementia. *Journal of Neuropsychology*, *10*(1), 130-142. <https://doi.org/10.1111/jnp.12062>

National Institute for Health and Care Excellence. (2017). *Intermediate care including reablement (NICE Guideline NG74)*. <https://www.nice.org.uk/guidance/ng74>

National Institute for Health and Care Excellence. (2018). *Dementia - assessment, management and support for people living with dementia and their carers (NICE Guideline NG97)*. [www.nice.org.uk/guidance/ng97](https://universityofexeteruk-my.sharepoint.com/personal/c_m_alexander_exeter_ac_uk/Documents/THESIS/Study%206%20HAPI%20development%20paper/v8%20supervisors%20edits/www.nice.org.uk/guidance/ng97)

Nelis, S. M., Clare, L., Martyr, A., Marková, I. S., Roth, I., Woods, R. T., Whitaker, C. J., & Morris, R. G. (2011). Awareness of social and emotional functioning in people with early-stage dementia and implications for carers. *Aging & Mental Health*, *15*(8), 961-969. <https://doi.org/10.1080/13607863.2011.575350>

NHS Digital. (2020). *The NHS digital SNOMED CT Browser*. <https://termbrowser.nhs.uk/>

Patterson, M. B., Mack, J. L., Neundorfer, M. M., Martin, R. J., Smyth, K. A., & Whitehouse, P. J. (1992). Assessment of functional ability in Alzheimer disease: a review and a preliminary report on the Cleveland Scale for Activities of Daily Living. *Alzheimer Disease & Associated Disorders*, *6*(3), 145-163. <https://doi.org/10.1097/00002093-199206030-00003>

Pfeffer, R. I., Kurosaki, T. T., Harrah, C. H., Jr., Chance, J. M., & Filos, S. (1982). Measurement of functional activities in older adults in the community. *Journal of Gerontology*, *37*(3), 323-329. <https://doi.org/10.1093/geronj/37.3.323>

Quinn, C., Morris, R. G., & Clare, L. (2018). Beliefs about dementia: development and validation of the Representations and Adjustment to Dementia Index (RADIX). *The American Journal of Geriatric Psychiatry*, *26*(6), 680-689. <https://doi.org/10.1016/j.jagp.2018.02.004>

Solfrizzi, V., Panza, F., Colacicco, A. M., D’Introno, A., Capurso, C., Torres, F., Grigoletto, F., Maggi, S., Del Parigi, A., Reiman, E. M., Caselli, R. J., Scafato, E., Farchi, G., Capurso, A., & Italian Longitudinal Study on Aging Working Group. (2004). Vascular risk factors, incidence of MCI, and rates of progression to dementia. *Neurology*, *63*(10), 1882-1891. <https://doi.org/10.1212/01.wnl.0000144281.38555.e3>

Solfrizzi, V., Panza, F., Imbimbo, B., D’Introno, A., Galluzzo, L., Gandin, C., Misciagna, G., Guerra, V., Osella, A., Baldereschi, M., Di Carlo, A., Inzitari, D., Seripa, D., Pilotto, A., Sabbà, C., Logroscino, G., Scafato, E., & Italian Longitudinal Study on Aging Working Group. (2015). Coffee consumption habits and the risk of mild cognitive impairment: the Italian Longitudinal Study on Aging. *Journal of Alzheimer's Disease*, *47*(4), 889-899. <https://doi.org/10.3233/JAD-150333>

The EuroQol Group. (1990). EuroQol - a new facility for the measurement of health-related quality of life. *Health Policy*, *16*(3), 199-208. <https://doi.org/10.1016/0168-8510(90)90421-9>

Wheatley, A., Brunskill, G., Dow, J., Bamford, C., Poole, M., Robinson, L., & the PriDem study team. (2022). The primary care annual dementia review: a qualitative study of the views and experiences of service users and providers. *medRxiv*. <https://doi.org/10.1101/2022.04.26.22274255>

Wilson, B. A., Greenfield, E., Clare, L., Baddeley, A. D., Cockburn, J., Watson, P., Tate, R., Sopena, S., & Nannery, R. (2007). *Rivermead Behavioural Memory Test - Third Edition (RBMT-3)*. Harcourt Assessment.

Wong, A., Black, S. E., Yiu, S. Y. P., Au, L. W. C., Lau, A. Y. L., Soo, Y. O. Y., Chan, A. Y. Y., Leung, T. W. H., Wong, L. K. S., Kwok, T. C. Y., Cheung, T. C. K., Leung, K. T., Lam, B. Y. K., Kwan, J. S. K., & Mok, V. C. T. (2018). Converting MMSE to MoCA and MoCA 5-minute protocol in an educationally heterogeneous sample with stroke or transient ischemic attack. *International Journal of Geriatric Psychiatry*, *33*(5), 729-734. <https://doi.org/10.1002/gps.4846>

Wong, A., Nyenhuis, D., Black, S. E., Law, L. S. N., Lo, E. S. K., Kwan, P. W. L., Au, L., Chan, A. Y. Y., Wong, L. K. S., Nasreddine, Z., & Mok, V. C. T. (2015). Montreal Cognitive Assessment 5-minute protocol is a brief, valid, reliable, and feasible cognitive screen for telephone administration. *Stroke*, *46*(4), 1059-1064. <https://doi.org/doi:10.1161/STROKEAHA.114.007253>
